# Scale‐dependent impact of land management on above‐ and belowground biodiversity

**DOI:** 10.1002/ece3.6675

**Published:** 2020-08-31

**Authors:** Eleonore L. Slabbert, Oliver Schweiger, Tesfaye Wubet, Antje Kautzner, Cornelia Baessler, Harald Auge, Christiane Roscher, Tiffany M. Knight

**Affiliations:** ^1^ Department of Community Ecology Helmholtz Centre for Environmental Research‐ UFZ Halle (Saale) Germany; ^2^ Institute of Biology Martin Luther University Halle‐Wittenberg Halle (Saale) Germany; ^3^ German Centre for Integrative Biodiversity Research (iDiv) Halle‐Jena‐Leipzig Leipzig Germany; ^4^ Department of Physiological Diversity Helmholtz Centre for Environmental Research‐ UFZ Leipzig Germany

**Keywords:** above‐ and belowground taxonomic groups, biodiversity, grassland management, rarefaction curve, scale‐dependent responses, spatial aggregation, species‐abundance distribution

## Abstract

1. Land management is known to have consequences for biodiversity; however, our synthetic understanding of its effects is limited due to highly variable results across studies, which vary in the focal taxa and spatial grain considered, as well as the response variables reported. Such synthetic knowledge is necessary for management of agroecosystems for high diversity and function.
2. To fill this knowledge gap, we investigated the importance of scale‐dependent effects of land management (LM) (pastures vs. meadows), on plant and soil microbe diversity (fungi and bacteria) across 5 study sites in Central Germany. Analyses included diversity partitioning of species richness and related biodiversity components (i.e., density of individuals, species‐abundance distribution, and spatial aggregation) at two spatial grains (α‐ and γ‐scale, 1 m^2^ and 16 km^2^, respectively).
3. Our results show scale‐dependent patterns in response to LM to be the norm rather than the exception and highlight the importance of measuring species richness and its underlying components at multiple spatial grains.
4. Our outcomes provide new insight to the complexity of scale‐dependent responses within and across taxonomic groups. They suggest that, despite close associations between taxa, LM responses are not easily extrapolated across multiple spatial grains and taxa. Responses of biodiversity to LM are often driven by changes to evenness and spatial aggregation, rather than by changes in individual density. High‐site specificity of LM effects might be due to a variety of context‐specific factors, such as historic land management, identity of grazers, and grazing regime.
5. *Synthesis and applications:* Our results suggest that links between taxa are not necessarily strong enough to allow for generalization of biodiversity patterns. These findings highlight the importance of considering multiple taxa and spatial grains when investigating LM responses, while promoting management practices that do the same and are tailored to local and regional conditions.

## Graphical abstract

We investigated the occurrence of scale‐dependent impacts of land management on above‐ and belowground biodiversity within managed grassland. Our results showed scale‐dependent patterns in response to land management to be the norm rather than the exception and highlight the importance of measuring species richness and its underlying components at multiple spatial grains. Outcomes provide new insight to the complexity of scale‐dependent responses within and across taxonomic groups.

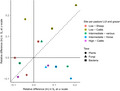

## 1 INTRODUCTION

It is critical to understand how land management (LM) influences the diversity of organisms if we are to maintain, and possibly restore biodiversity and the ecological functions that it provides. Response patterns to these factors depend on the focal taxonomic group considered and the spatial grain of measurement. Taxa are known to respond differently to the same LM gradient (Gossner et al., 2016; Penone et al., 2018), likely due to their different generation times, dispersal abilities, and other life‐history characteristics. Effects of LM on biodiversity can become less prominent with increasing spatial grains as environmental variability created by LM decreases through spatial averaging at larger scales of investigation (Levin, 1992) and other environmental factors, such as climate, can increase in importance (Carl, Doktor, & Schweiger, 2016). Therefore, our understanding of the effects of LM on biodiversity would be improved by studies that consider both multiple taxa and different spatial grains.

The scale‐dependent effects of LM on observed species richness depend on their effects on its underlying biodiversity components, namely (a) the density of individuals (i.e., species abundance), (b) their relative abundances or the evenness of the community (i.e., species‐abundance distribution), and (c) the spatial aggregation of species (Chase et al., 2018; Simons et al., 2017). For example, if LM primarily reduces the density of individuals, the impact on species richness may only be evident at smaller spatial grains since fewer species are observed when there are fewer individuals. However, with increasing grain, the chances of observing at least one individual of rarer species increases. LM may also alter the evenness of communities by changing the availability of specific resources. For instance, nutrient input and/or high access to light in grasslands with intense LM can result in the dominance of species adapted to these conditions (Hülbera et al., 2017; Ignatavičius, Sinkevičius, & Ložytė, 2013). Thus, most individuals sampled at small grains would be those of the dominant species, whereas rare species would be observed at larger grains. Finally, LM can affect the spatial aggregation of species, for example, by altering the heterogeneity of the habitat, presence of different microsites, and by influencing the dispersal of propagules (Baltzinger, Karimi, & Shukla, 2019; Tälle et al., 2016). For instance, a decrease in habitat heterogeneity by specific LM practices has been shown to homogenize biotic communities (Allan et al., 2014; Gossner et al., 2016; Hendrickx et al., 2007). In this case, LM effects on biodiversity would become more apparent at larger spatial grains.

Furthermore, it has also been shown that within an ecosystem different species groups can react differently to environmental drivers and that these differences can be scale‐dependent (Gossner et al., 2016; Penone et al., 2018; Schuldt et al., 2015). For example, local species richness of belowground soil biota are less or even positively affected by intense agricultural land use in comparison with aboveground taxa which show a more pronounced negative response (Allan et al., 2014; Gossner et al., 2016). However, at larger spatial scales, responses are more similar between above‐ and belowground taxa (Gossner et al., 2016). Yet, it remains unclear which biodiversity components (i.e., density of individuals, community evenness, spatial turnover) are causing these taxa‐specific scale‐dependent responses.

To investigate the scale‐ and taxa‐specific effects of LM on biodiversity and the underlying components, we considered seminatural grasslands in Central Germany under different LMs (pastures vs. meadows). Seminatural grasslands have formed due to historic land use practices and are some of the most species‐rich habitats in Europe (Hönigová et al., 2012; Tälle et al., 2016). Seminatural grasslands are of value not only for their rich biodiversity of plant and animal species, but also as productive agroecosystems that provide an array of ecosystem functions and services (Hönigová et al., 2012; Ignatavičius et al., 2013). Traditional management of these grasslands using either low‐intensity mowing and grazing is known to support high biodiversity, and it is unclear if one LM type promotes more biodiversity than the other. Increasing the intensity of either LM type, for example, through increased fertilization, mowing frequency or grazing intensity, is well‐known to have negative consequences for biodiversity (Dahlström, Iuga, & Lennartsson, 2013; Ignatavičius et al., 2013; Socher et al., 2012; Tälle et al., 2016).

There is high variation across studies in the effects of grassland LM on biodiversity. A meta‐analysis by Tälle et al. (2016) found that, within pasture‐meadow comparisons, there was only a marginally more positive effect of pasture management in comparison to meadows in species richness of multiple taxa (e.g., insects, plants, earthworms, and spiders). Further analyses found effects to vary by grassland characteristics (e.g., grassland types) and many other factors that vary between studies, such as context‐specific differences between different continents, grazer identities, and forms of intensification. The meta‐analysis did not explicitly consider the spatial grains of the study, or underlying biodiversity components, which might also explain variation in richness responses to LM. The few studies on the effects of LM on soil microbial communities also show variable results. Some reporting significant shifts in community composition and structure (Patra et al., 2005; Wang et al., 2013), while other studies have found LM to have little to no effect (Bardgett & McAlister, 1999; Harold et al., 2014; Penone et al., 2018).

In the present study, we specifically compare grasslands managed for livestock grazing to those managed for hay production with the aim of explicitly investigating the importance of scale‐dependent responses of multiple taxa to these LM types. Our study considers five sites, and each site has replicate grasslands of each LM type. The meadow management of all sites is similar, but the pasture management includes a variety of contexts (e.g., differences in grazing intensities and grazer species). Each study site provides a test to determine how LM influences species richness across taxa and spatial scales (i.e., α‐ (1 m^2^) and γ‐scales (16 km^2^)), and which component of biodiversity (density of individuals, evenness, and spatial turnover) is most affected by LM. Across all five sites, we can assess whether there is any generality in these responses to LM, or if biodiversity conservation will require consideration of other aspects of the management context.

We expect scale‐dependent effects of LM on biodiversity. Since grazers have localized disturbances (e.g., by trampling), we predict more prominent LM effects at the α‐ and ß‐scale resulting in scale‐dependent responses in species richness were pastures have higher richness in comparison with meadows. Due to grazer selectivity, we also expect stronger impacts of evenness and spatial aggregation on local species richness and turnover. Second, we expect that more closely linked taxa will have similar response patterns to LM (Bever, Westover, & Antonovics, 1997; Neuenkamp et al., 2018). For example, belowground soil microbe communities that are more directly connected with plant communities (e.g., soil fungi through mutualistic and symbiotic interactions) are expected to resemble plant responses to LM, while organisms with weaker links to plants (e.g., soil bacteria) should respond more independently of LM (e.g., see Hedlund et al., 2004).

## 2 METHODS AND MATERIALS

### 2.1 Study area

We selected five study sites which form part of the Terrestrial Environmental Observatories (TERENO) (Zacharias et al., 2011). These sites are also part of the German and European Long‐term Ecological Research networks. The latter being initiated in 2009 as part of the former EU FP5 GREENVEINS project (Billeter et al., 2008). Each site is 4 km by 4 km and represents typical agro‐ecological landscapes in Central Germany and comparable landscapes across Europe. Sites differ in their extent of agricultural intensity, land management practices, and biophysical characteristics (e.g., mean annual precipitation and temperature; topography, see Frenzel, Everaars, & Schweiger, 2016), including soil chemical properties (Table S1). Unfertilized grasslands, managed predominantly for livestock grazing (pastures) or hay production (mown meadows, henceforth referred to as “meadows”), were identified within each site as the focal system of our study.

The placement of LM types within each site by farmers might be not at random, but based on local site conditions, such as topography or local soil conditions, which could confound our results of LM effects on biodiversity and, moreover, restrict a farmer's flexibility in decision making. We investigated this possibility and found that pastures and meadows did not differ consistently across the different sites in chemical soil properties (Figure S1, Table S2), but did differ in some topology features (e.g., slope) (Figure S2, Table S2).

The initial study design was balanced and nested with three grasslands per LM type per study site, each with a randomly placed sampling plot of 10 m × 10 m. Plots were subdivided into subplots of 1 m^2^ from which 10 were randomly selected for sampling plants and soil microbes. Due to in‐field limitations and more detailed records from farmers on field‐specific management practices, the final dataset consisted of 270 subplots, 120 from meadows and 150 from pastures (Table 1) leading to a slightly imbalanced sampling design. All grasslands were in use as the respective LM type for at least the last 10 years. Meadows had similar mowing frequencies (once or twice), but the grazing intensities of the pastures differed (Table 1). We summarize the land use intensity (LUI) of pastures at each site by their grazing intensity per plot. Specifically, we used equivalent livestock units per hectare per annum standardized across the different grazer species (horse, cattle, sheep, mixed; Table 1) and categorized them to low, intermediate, and high intensity levels. With five sites, we do not have the statistical power to test how grazing intensity influences biodiversity responses to LM across spatial scales. However, these site categories do help with data visualization and discussion.

**TABLE 1:** Summary of land management (LM) and pasture grazing intensity of unfertilized seminatural grasslands managed as pastures or meadows in the Terrestrial Environmental Observatories, Central Germany

Study site*	Location	Meadow		Area (ha)	Pasture**			Area (ha)	Total
		No. of subplots	Mowing frequency/a		No. of subplots	Grazing intensity/a	Grazing intensity category and livestock identity		
Harsleben	51.8423°N, 11.0753°E	20	1	2.00–6.22	40	0.43	Low‐sheep	2.29–67.00	60
Siptenfelde	51.6491°N, 11.0526°E	30	1	11.96–23.95	20	0.36–0.48	Low‐cattle	2.53–6.76	50
Friedeburg	51.6177°N, 11.7096°E	30	1–2	0.84–1.93	30	0.23–0.9	Intermediate‐various (cattle, goats, horses)	1.07–1.72	60
Wanzleben	52.0803°N, 11.4518°E	30	1–2	3.72–7.44	30	0.75–0.83	Intermediate‐horses	4.03	60
Greifenhagen	51.6329°N, 11.4340°E	10	2	1.96	30	0.99–2.877	High‐cattle	0.45–5.44	40
Total		120			150				270

Note

*Site names refer to the nearest large village.

**Average mowing and grazing intensities per annum of the respective LM types, as well as different grazers present on pastures are indicated. Grazing intensity was standardized as livestock units per hectare per annum (LSU/ha/a). Three grazing intensity categories (low, intermediate, and high) were allocated to the sites based on the LSU/ha/a for increased ease of visualization and discussion of results.

### 2.2 Data collection and processing

Aboveground vascular plants and belowground, fungi and bacteria, were sampled during summer 2014. The finest spatial resolution was at subplot level (α‐scale of 1 m^2^), which was pooled to reach the γ‐scale at site level (16 km^2^), with turnover between them as ß‐diversity. We did not consider the intermediate grain (plot), but rather focused on the extremes of the scale gradient (i.e. subplot level and site level). Sampling included species richness and species abundances within the respective taxonomic groups per subplot. All vascular plant species were identified to species level, and their cover was visually estimated to the nearest percentage as a proxy for abundance. Nomenclature was cross referenced and updated according to “The Plant List” (2013). Soil microbial communities were sampled per subplot using a standard composite sampling approach whereby 5 soil cores of ca. 6 cm diameter to 10 cm depth (after removal of loose organic matter) have been collected and then pooled in‐field and sieved to 2 mm. Of the pooled subplot sample, ten grams of the soil sample was flash‐frozen on dry‐ice for microbial analysis. A total of 270 soil samples were collected for further processing. An overview of the plant, fungi, and bacterial data is provided in the Table S3.

### 2.3 DNA extraction, amplicon library preparation, and Illumina MiSeq sequencing

Soil microbial genomic DNA was extracted from 0.25 g of each soil sample using a PowerSoil DNA Isolation Kit (MO BIO Laboratories Inc.). DNA yields were quantified with a NanoDrop ND‐8000 spectrophotometer (Thermo Fisher Scientific), adjusted to 10–15 ng/μl, and stored at −20°C. The V4 bacterial 16S rRNA gene fragment was amplified using the universal primer pair 515f and 806r (Caporaso et al., 2010) with Illumina adapter sequences. The PCR condition was initial denaturation at 95°C for 3 min, 25 cycles of denaturation at 98°C for 20 s, annealing at 55°C for 15 s, elongation at 72°C for 15 s, and a final extension at 72°C for 5 min. To generate the fungal amplicon library, seminested PCRs were performed, starting with amplification of the fungal ITS rDNA region using the primer combination ITS1F (Gardes & Burns, 1993) and ITS4 (White et al., 1990). The PCR thermo‐cycle conditions were as follows: initial denaturation at 95°C for 5 min, 10 cycles of denaturation at 98°C for 20 s, annealing at 50–60°C for 15 s (−1°C per cycle), followed by elongation at 72°C for 15 s and 2 cycles of denaturation at 98°C for 20 s, annealing at 50°C for 15 s, followed by elongation at 72°C for 15 s. The final extension was carried out at 72°C for 5 min. The ITS2 region was subsequently amplified using 1:10 diluted products of the first PCR and the primer pair fITS7 (Ihrmark et al., 2012) and ITS4 (White et al., 1990). PCR was performed under the following conditions: initial denaturation at 95°C for 5 min, 25 cycles of denaturation at 98°C for 20 s, annealing at 56°C for 15 s, followed by elongation at 72°C for 15 s, and a final extension at 72°C for 5 min. All PCRs were conducted using the proofreading Kapa Hifi polymerase (Kapa Biosystems). Paired‐end sequencing of the equimolar pooled fungal and bacterial amplicon libraries was performed using a MiSeq Reagent kit v3 (2 × 300 bp) on an Illumina MiSeq platform (Illumina Inc.). The raw sequence datasets were deposited in the National Center for Biotechnology Information (NCBI) Sequence Read Archive (SRA) under the accession PRJNA563995.

### 2.4 Bioinformatic analysis of the microbial datasets

Sequences from individual samples were de‐multiplexed by the Illumina MiSeq Reporter software package v2.5.1.3 and then processed using custom bash scripts on a high‐performance computing cluster following the workflow presented in Schöps et al. (2018). Briefly, paired‐end reads were merged using PANDASeq v2.8. (Masella, Bartram, Truszkowski, Brown, & Neufeld, 2012) and the assembled reads were quality filtered using MOTHUR v1.39.5. Chimeric sequences were detected using the UCHIME algorithm in de novo mode as implemented in MOTHUR (Schloss et al., 2009). Reads from each sample were pooled, dereplicated, and sorted by decreasing abundance and preclustered. The cd‐hit‐est v4.6.1 algorithm (Fu, Niu, Zhu, Wu, & Li, 2012) was used to cluster sequences into operational taxonomic units (OTUs) at a similarity threshold of 97%. The representative sequences were classified against the UNITE database v7 (Köljalg et al., 2013) for fungi and against the SILVA database v128 (2016‐11‐28; Quast et al., 2012) for bacterial sequences using the Bayesian classifier as implemented in MOTHUR (Schloss et al., 2009). Rare OTUs were removed from the dataset to remove the impact of potential sequencing artifacts, OTU inflations and to reduce excessive variability due to extremely low occurrences. The data matrix was filtered to only include OTU’s that occurred more than 5 times in at least 1% of the dataset using the “phyloseq” package (McMurdie & Holmes, 2019).

### 2.5 Statistical approach

To investigate scale‐dependent responses of the three taxonomic groups (plants, fungi, bacteria) to LM, we used the “measures of biodiversity” package (“mobr”; McGlinn et al., 2019) within R (R Core Team, 2019) to calculate biodiversity indices for α‐, γ‐, and β‐diversity and followed the analytical framework as outlined in Chase et al. (2018) and McGlinn et al. (2019). In addition to overall abundance (i.e., % cover of plants and OTU reads of soil microbes) (*N*) and observed species richness (*S*), we also calculated rarefied richness (*S*_n_) investigating whether LM effects on biodiversity were solely caused by differences in N or have density‐independent effects on species richness. For instance, an effect of LM on *S* but not *S*_n_ is interpreted as a sole effect of *N*. Additionally, a measure of community evenness (*S*_PIE_) tests whether LM changes the shape of the species‐abundance distributions at α‐scale and γ‐scale. Comparisons of responses of *S*_PIE_ with that of *S*_n_ allow to assess whether the effects of LM on species richness are direct or rather indirectly caused by changes in evenness. At α‐scale, species richness was rarefied to the minimum total number of individuals within a subplot across LM type using individual‐based rarefaction curves, while for γ‐scale, this minimum was multiplied by the number of replicates per LM. The slope at the base of the individual‐based rarefaction curves yields the probability of intraspecific encounter (PIE) (i.e., an evenness metric) (Hurlbert, 1971) and is the equivalent to 1—Simpson's index (Jost, 2006). For better comparisons to *S* and *S*_n_, we converted PIE to an effective number of species (*S*_PIE_) (i.e., the number of equally abundant species needed to reach the given species richness) (e.g., Hill, 1973; Jost, 2006, 2007). *S*_PIE_ captures changes in community evenness, with a particular weight on common species in comparison with changes in *S*, which gives equal weights to all species (McGlinn et al., 2019). *S*_PIE_ is based on species accumulation curves which cover density, evenness, and (implicitly) spatial extent. Since *S*_PIE_ is calculated as slope at the basis of these species accumulation curves, it is independent of both species pool and spatial scale. This ensures an unbiased estimation of *S*_PIE_ at α‐ and γ‐scales, except under significantly altered community aggregation (Chase et al., 2018; McGlinn et al., 2019).

Disentangling the different underlying mechanism determining the response of species turnover (β‐diversity) to LM follows in principle the same rationale than for α‐diversity and γ‐diversity, that is, comparing responses of *S*, *S*_n_, and *S*_PIE_. However, since the analyses of α‐diversity and γ‐diversity indicated a predominant role of evenness, we focused on β‐diversity based on *S*_n_ and *S*_PIE_. We use a multiplicative β‐diversity metric to determine β‐S_n_ and β‐S_PIE_ (Whittaker, 1960). The influence of spatial aggregation (i.e., intraspecific clustering) can be disentangled using β‐S_n_, calculated from the same n (i.e., minimum total number of individuals within a subplot) for α‐ and γ‐scales to control for density and species‐abundance distribution effects (McGlinn et al., 2019). A high β‐S_n_ relates to an increased spatial aggregation of common and rare species, while controlling for the effect of *N*, while β‐S_PIE_ is more representative of aggregation among common species. By comparing β‐S_n_ and β‐S_PIE_, we identify the impact of turnover in evenness on spatial aggregation in comparison with turnover of species. A summary table adapted from Chase et al. (2018) of biodiversity metrics and their descriptions are in the supplementary material (Table S4).

We assessed the effect of LM (pasture vs mowing) on S and the different components of biodiversity separately for each site and taxonomic group. The effect sizes of LM were summarized as relative differences (i.e., log‐response ratios) (Hedges, Gurevitch, & Curtis, 1999) and were then quantitatively compared with analyses of variance and permutation tests (perm = 199) (McGlinn et al., 2019). At α‐scale, we used one‐way analyses of variance (*F*‐statistic) to compare observed LM differences to the null hypothesis of no difference. At the γ‐scale, where there is only one value per treatment, the average relative difference between treatments was compared to a permuted distribution to determine an equivalent p‐value statistic. Permutation (perm = 199) for γ‐scale took place on data pooled across LM types. The null distribution was determined by calculating the difference in diversity indices for the LM types per permutation (Chase et al., 2018). Sampling imbalances across LM types were accounted for by standardizing sampling effort by a repeated resampling procedure across the LM comparisons as needed at three of the five sites, that is, by repeatedly limiting the number of subplots per LM type to the minimum number available across LM types. The number of standardized replicates was determined by the total number of unique plot combinations possible without replacement. Replicated metrics and test statistics from this standardization were averaged using the R package “harmonicmeanp” (Wilson, 2019).

## 3 RESULTS

### 3.1 General overview

**FIGURE 1:**
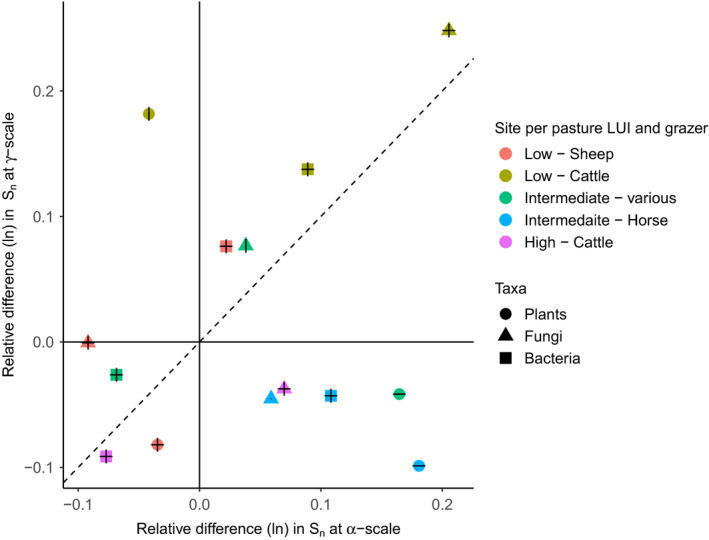
Scale‐dependent impact of land management (pasture vs. meadow) observed as a change in the log‐response ratio (Relative difference (ln)) in rarefied species richness (Sn) at the α‐scale (subplot level, 1 m^2^) and γ‐scale (site level, 16 km^2^) for above‐ (i.e., plants, circles) and belowground taxa (i.e., soil fungi and bacteria, triangles and squares, respectively). The log‐response ratio between management types was calculated with meadows as reference, thus positive values indicated that S_n_ is higher in pasture management. Horizontal and vertical bars indicate significant differences (*p* < .05) in S_n_ between LM types based on the ANOVA and permutation tests, for α‐ and γ‐scales, respectively. The dashed 1:1 line indicates no scale dependence. Sites are color coded according to pasture land use intensity (LUI) calculated as livestock units per hectare per annum, see Table 1 for more details. The plant community comparison at the highest pasture LUI was excluded as this distorted the scale for other comparisons (α‐ & γ‐scale S_n_, −6.4 and −20.0, respectively) (see Figure S1)

**FIGURE 2:**
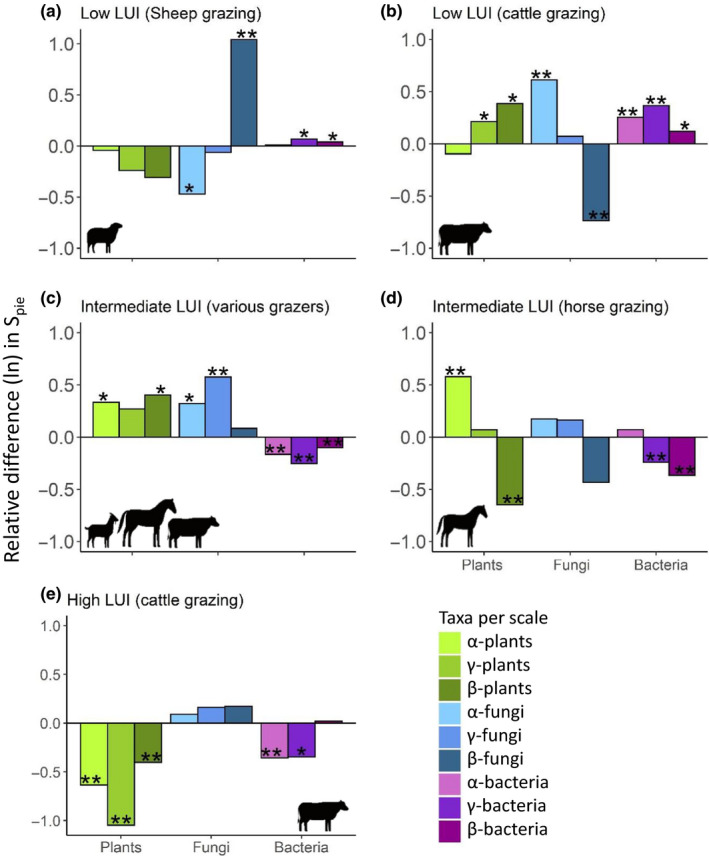
Scale‐dependent impact of land management (pasture vs. meadow) on the log‐response ratio (Relative difference (ln)) of effective number of species (S_PIE_) for above‐ (i.e., plants, green) and belowground taxa (i.e., soil fungi and bacteria, blue and purple, respectively). The log‐response ratios between management types were calculated with meadows as reference at α‐scale (subplot level, 1 m^2^) and γ‐scale (site level, 16 km^2^), and β‐diversity (scales indicated from lightest to darkest hue) per taxa. Positive values of α‐ and γ‐S_PIE_ indicate that pastures have more even communities compared to meadows, while a positive β‐S_PIE_ is representative of higher turnover among common species in pastures. Asterisks’ indicate significance differences (*p < .05; **p < .01; ***p < .001) between management types based on ANOVA and permutation tests, for α‐ and γ‐scales, respectively. Sites are labeled according to pasture land use intensity (LUI) calculated as livestock units per hectare per annum, see Table 1 for more details

We identified scale‐dependent responses of species richness (S) to land management (LM) for all species groups (Figure S3). These effects were only partly defined by differences in overall abundance (*N*) and remained qualitatively the same for rarefied richness (*S*_n_) (Figure 1, Table S5). Responses of *S* and *S*_n_ to LM were highly site‐specific and often not consistent within or across taxa. The underlying biodiversity component resulting in these responses was, however, often driven by a change in species evenness (α‐ and γ‐S_PIE_), and by turnover across subplots in the identity of the dominant species (β‐S_PIE_) (Figure 2, Table S5). In general, pasture LM increased *S*_n_ at sites with intermediate levels of LUI, while at sites with the lowest and highest pasture LUI, the meadow management had higher *S*_n_ (Figure 1, bottom left quadrant), especially for plants under the highest grazing LUI (Figure S4). Higher *S*_n_ in pastures compared to meadows was more common at the α‐scale, but also present at the γ‐scale, and often at both (e.g., bacteria and fungi at the low‐cattle site; Figure 1).

### 3.2 Species richness

The observed responses of *S*_n_ to LM included many reversals in direction across scales, as represented by points falling within the top left‐ and bottom right‐hand quadrants of Figure 1. Qualitative scale‐dependent responses, that is, with a significant reversal across both scales, included the fungal communities at the sites with low‐sheep and high‐cattle LUI and in the bacterial community at the site with intermediate‐horse LUI (Figure 1). The other scale‐dependent responses only had significant LM impact (*p* < .05) at one of the scales for specific taxa groups. These included plants at the sites with intermediate LUI with a LM response at only the α‐scale (Figure 1, bottom right‐hand quadrant); while plants at the low LUI cattle grazed site only had a LM response at the γ‐scale (Figure 1, top left‐hand quadrant).

The frequency and direction of the response of *S*_n_ to LM was consistent across scales for specific taxa groups at some sites (e.g., plants and bacteria at high‐cattle LUI, and bacteria and fungi at low‐cattle LUI), but also varied across scales (e.g., fungi at the low‐sheep and high‐cattle site; and plants and bacteria at the intermediate‐horse site) and between the respective taxonomic groups at some sites (e.g., plants and bacteria at the low‐sheep site; and bacteria and fungi at intermediate‐various site) (Figure 1). Within a few sites, all taxa responded similarly to LM (e.g., pasture management resulted in higher γ‐S_n_ at the low‐cattle site, while the LM resulted in lower γ‐S_n_ at the intermediate‐horse‐grazed site).

Bacteria had a significant change in *S*_n_ as response to LM across both scales for all five sites, although with less pronounced scale dependence (Figure 1). Pasture management generally increased α‐ and γ‐S_n_ of bacteria at the lower LUI, and decreased S_n_ at the intermediate to highest LUI, with the exception of the horse‐grazed sites α‐S_n_. In comparison, plants and fungi were only moderately less responsive at the respective scales of investigation, and also had site‐specific LM outcomes on S_n_. Pasture management decreased α‐ and γ‐S_n_ for plants at sites with the highest LUI (Figure S4) and low LUI, with cattle and sheep grazing, respectively (Figure 1). While at other sites, with low‐to‐intermediate LUI grazing, plant *S*_n_ increased at both scales. The impact on fungal S_n_ at both scales was the least consistent, with the direction of impact occasionally being in reverse across scales as highlighted before (Figure 1).

### 3.3 Biodiversity components resulting in scale‐dependent responses

Pasture management mostly increased N, with the exception of plants at the low‐cattle site (Figure S5). The magnitude of change in N across LM types was, however, much lower than the contribution of altered community evenness of common species (α‐ and γ‐S_PIE_) or the differences in their spatial turnover (β‐S_PIE_) (Figure 2). The direction of altered dominance among common species between the LM types (Figure 2) often reflected the scale‐related responses observed in *S*_n_ (Figure 1). For example, at the low‐cattle site, pasture management increased plant richness at the γ level but not α level (Figure 1), and plant species evenness was also higher with pasture management at the γ level, but not α level (Figure 2).

For all taxonomic groups, changes in community turnover across the two LM types were caused by species turnover (β‐S_n_) to a lesser extent, while changes in evenness (β‐S_PIE_) contributed the most with some consistency within the respective sites between taxa (Figure 2a,b,d,e, β‐S_PIE_ and Figure S6). The relative contribution of altered β‐diversity to the change in *S*_n_ was usually lower than that of *S*_PIE_, with the exception of fungi at the lower LUI (Figure 2a,b). Here, β‐S_PIE_ of fungal species either increased (Figure 2a) or decreased (Figure 2b) much more than at other sites or for the other taxa groups. Interestingly, LM occasionally altered β‐S_PIE_ in the opposite direction than its impact on *S*_PIE_ (Figure 2a,b,d).

## 4 DISCUSSION

### 4.1 General overview

Scale‐dependent responses to LM were evident across both above‐ and belowground taxonomic groups and for all sites, indicated by no LM comparisons falling on the 1:1 line in Figure 1. Our prediction that pasture LM would increase species richness (*S* and *S*_n_) was mostly supported with only a few exceptions. However, the scale‐dependent patterns within and across taxa groups were less consistent than expected. In 50% of the cases, we observed a reversal of LM impacts across the α‐ (1 m^2^) and γ‐ (16 km^2^) scale, but the α‐scale impact was not necessarily consistently more prominent as we initially predicted. Changes in *S*, irrespective of the directionality, were primarily driven by LM altering community evenness of common species (*S*_PIE_), as well as the spatial aggregation of both common and rare species (β‐*S*_n_ and β‐*S*_PIE_), rather than changes in species abundance (*N*). Considering our second hypothesis, regarding similarity of scale‐dependent LM responses within closer linked taxa groups, our results showed no clear consistency for plants and fungi. LM effects were inconsistent among the sites, suggesting that context‐specific factors, such as grazing intensity and grazer identity, might be important. Further, other factors, such as the time and seasonality of grazing or other unmeasured abiotic conditions, may influence biodiversity responses to LM. Our results suggest the need for studies that explicitly sample a variety of context‐dependent factors that vary across sites.

### 4.2 Scale dependency of LM, and the impact of grazing intensity and grazer identity

The response of *S*_n_ to LM was highly scale‐dependent, and the direction of the effect varied at different grazing intensities. Pasture management often resulted in higher species richness at the α‐ and γ‐scales at sites with intermediate grazing intensities, while the positive effect of pasture management was in reverse at the lowest and highest grazing intensity sites. These LM results are consistent with the idea that disturbances of intermediate intensity and frequency allow for higher diversity through creating habitat heterogeneity at γ‐scale and modulating competition among species at the α‐scale (Connell, 1978). Our results coincide with some of the studies in the meta‐analyses by Tälle et al. (2016), several of which found grazing, especially in central Europe, to favor higher species richness in grasslands.

The higher *S*_n_ in pastures in comparison with meadows was due to an increase in pasture communities’ evenness and species turnover, especially under certain low‐to‐intermediate grazing intensities. This suggests that the positive impact of pasture management could be a consequence of higher habitat heterogeneity, which likely promotes higher species coexistence and spatial aggregation of habitat specialists. This increase in evenness of pasture communities is in contrast to other grassland studies in the meta‐analyses by Tälle et al. (2016) that found mowing, rather than grazing, to increase community evenness. Another noteworthy finding includes the observation that diversity patterns were more strongly driven by a change in common species, and not only due to a loss of rare species. Observations that could be explained by the “niche differentiation hypothesis” (Connell, 1978); with more diversity of habitat niches, more species can coexist as species can spatially be arranged according to their resource needs. Contrastingly, grazing reduced local species richness for most taxa groups at two sites: the pasture with low‐sheep grazing intensity and the site with high‐cattle grazing intensity. Here, the negative impact on the respective communities was a result of grazing promoting the dominance of only a few species, possibly by selecting plant species with a high tolerance for grazing, excessive trampling, or both (e.g., *Lolium perenne* and *Festuca rubra*). These results suggest even higher site specificity than found by Tälle et al. (2016).

The high‐site specificity of the LM impacts can be due to a variety of site‐specific factors, such as historic LM, identity of the grazers, grazing regime, soil properties, or topography. Legacy effects of historic LM practices on our grasslands could be resulting in less consistent scale‐dependent effects than expected across scales and taxa. It is known that past landscape structure and long‐term LM and LUI of an area have a significant role in shaping current biodiversity patterns (Gustavsson, Lennartsson, & Emanuelsson, 2007; Poschlod, Kiefer, Tränkle, Fischer, & Bonn, 1998). Continuity of historic LM, for example, has been found to have lasting effects on the local communities by determining current species pools (e.g., of grassland plants) (Eriksson, Eriksson, & Berglund, 1995; Gustavsson et al., 2007). The study by Gustavsson et al. (2007) found land use of 200 years ago to be a better predictor of biodiversity patterns for both plants and soil microbes than current land use. This suggests LM to have had a time‐lagged effect on these communities. Comparable soil chemical properties across LM types at certain sites hint at similar historic fertilizer applications that's effects are still evident. Unfortunately, a lack of historic data prevented us from investigating such potential legacy effects.

A second factor influencing the high‐site specificity could be grazer identity, despite previous studies that have shown it to be of lesser importance than grazing intensity in shaping grassland communities (Stewart & Pullin, 2008). The occurrence of site‐specific scale‐dependent responses, especially for fungi and to some degree plants, suggests that grazer identity may potentially be a prominent factor. Different grazers alter the local microclimate and habitat heterogeneity in distinct ways while also impacting the dispersal patterns of propagules (e.g., via endo‐ or epizoochory) (Baltzinger et al., 2019; Golan & Pringle, 2017). For instance, the amount and effectiveness of dispersal is correlated with body size (i.e., volume of biomass they consume) and other properties linked to grazer identity, such as feeding habit, behavior, and fur or hair characteristics (Baltzinger et al., 2019). The impact of grazers on community dynamics could seem counterintuitive in both creating higher habitat heterogeneity that leads to higher coexistence (i.e., high β‐diversity), while also facilitating dispersal that would lower β‐diversity. Our results, contest this, showing that the overall “net” outcome can still be an increase in species richness. In contrast, the less frequent removal of biomass through mowing results in more homogeneous habitat conditions, higher nutrient inputs, and increased competition for light (Hülbera et al., 2017; Ignatavičius et al., 2013). These conditions could result in a shift in species composition, with higher intraspecies competition and increased dominance of species tolerant to these conditions, as our results suggest for instance in the plants and bacterial communities of the high grazing intensity site. Another factor influencing high‐site specificity could be grazing regime, for example, continuous versus rotational grazing and extensive versus intensive grazing. Although it is not statistically considered in detail here, these management decisions and the movements of grazers between fields would further influence observed biodiversity patterns and the size of the species pool influencing the richness of the local communities (Poschlod et al., 1998).

We did not find consistent differences in soil chemical properties (Figure S1, Table S2) and only a slight but expected preference for pastures at steeper slopes (Figure S2, Table S2). Thus, soil conditions might not be responsible for site specificity, but on the other hand, this indicates greater flexibility of independent management decisions, within some topographical boundary conditions, strengthening the relevance of our results in terms of conservation.

### 4.3 Scale‐dependent responses within and across taxa.

The LM response of plants and fungi, as closer associated taxa, was not remarkably more similar to each other in comparison with bacteria as we hypothesized. Our findings are in contrast to previous studies that found more linked LM responses (Hedlund et al., 2004) or consistent responses for above‐ and belowground taxonomic groups to LM (Gossner et al., 2016; Simons et al., 2017). Our results suggest that these trends are not as simple when multiple spatial grains and highly variable sites are considered. Similarly, Schuldt et al. (2015) found that fungi and bacteria had distinct scale‐dependent response rates in species turnover. Together, these results also point to higher complexity of scale‐dependent responses of belowground soil microbiota to environmental factors, and advocates for more scale‐explicit investigation of soil microbial communities. Similar site‐specific factors, as outlined above, could be causing the low consistency of LM responses within and across taxa groups. Legacy effect's on the taxa groups could, for instance, be temporally staggered (e.g., due to different turnover rates). Thus, plant communities, for example, could be reflecting LM of a few centuries ago (Gustavsson et al., 2007), while microbial communities, with shorter generation times, could be more representative of current LM responses (Felske & Akkermans, 1998).

## 5 CONCLUSIONS

Our results highlight that scale‐dependent patterns in responses to land management (LM) are the norm rather than the exception. This emphasizes the importance of investigating the underlying components resulting in these patterns. Despite clear links and interactions influencing diversity patterns of above‐ and belowground taxa, our findings suggest these links to not be strong enough for generalization of biodiversity patterns. Furthermore, that the simple dichotomy between the two LM types (here pasture and meadow management of grasslands) fails to accurately consider the context specificity of scale‐ and taxa‐dependent responses to LM.

Our findings affirm existing management recommendations advocating low‐to‐moderate grazing intensities for promoting biodiversity, through creating habitat heterogeneity, and warn against too high grazing intensities which can reduce species richness. Our study provides a first step in our understanding of the management that might promote biodiversity of multiple taxa at multiple spatial scales, but the context dependency highlights the need for more studies that consider multiple scales and taxa within a variety of contexts (e.g., grazing regime, historical land use). For the time being, we recommend that overarching policies, such as the European Common Agricultural Policy (CAP), advance agro‐biodiversity conservation by having a framework that allows for local adaptation of management regimes, and prioritizes conservation of multiple taxa, across multiple spatial grains.

## CONFLICT OF INTEREST

The authors have no conflicting interests to declare.

## AUTHOR CONTRIBUTION

**Eleonore L. Slabbert:** Conceptualization (lead); Data curation (lead); Formal analysis (lead); Investigation (lead); Methodology (lead); Project administration (lead); Software (lead); Validation (lead); Visualization (lead); Writing‐original draft (lead); Writing‐review & editing (lead). **Oliver Schweiger:** Conceptualization (equal); Formal analysis (equal); Funding acquisition (equal); Methodology (supporting); Project administration (supporting); Supervision (equal); Validation (equal); Visualization (supporting); Writing‐original draft (equal); Writing‐review & editing (equal). **Tesfaye Wubet:** Conceptualization (equal); Formal analysis (supporting); Investigation (supporting); Methodology (supporting); Resources (equal); Supervision (supporting); Validation (supporting); Writing‐original draft (supporting); Writing‐review & editing (supporting). **Antje Kautzner:** Conceptualization (equal); Data curation (equal); Investigation (lead); Project administration (equal); Writing‐original draft (supporting); Writing‐review & editing (supporting). **Cornelia Baessler:** Conceptualization (equal); Funding acquisition (equal); Investigation (supporting); Project administration (supporting). **Harald Auge:** Conceptualization (equal); Investigation (equal); Validation (supporting); Visualization (supporting); Writing‐original draft (supporting); Writing‐review & editing (supporting). **Christiane Roscher:** Conceptualization (equal); Investigation (equal); Validation (supporting); Visualization (supporting); Writing‐original draft (supporting); Writing‐review & editing (supporting). **Tiffany M. Knight:** Conceptualization (lead); Formal analysis (supporting); Methodology (equal); Supervision (equal); Validation (equal); Visualization (supporting); Writing‐original draft (equal); Writing‐review & editing (equal).

## Supporting information

Appendix S1

## Data Availability

Plant data is archived on PANGAEA (Kautzner, Auge, Roscher, Baessler & Slabbert, 2020 https://doi.org10.1594/PANGAEA.919343) and the soil microbial data on the National Center for Biotechnology Information (NCBI) Sequence Read Archive (SRA) under the accession PRJNA563995 (Kautzner, Baessler, Auge, Roscher & Wubet, 2020).
