# Supplementary material for: Scale‐dependent impact of land management on above‐ and belowground biodiversity
Source: Ecol Evol. 2020 Aug 31;10(18):10139–49. doi: 10.1002/ece3.6675 (PMC7520218; doi:10.1002/ece3.6675)
Supplement: Supplementary file 1 — Appendix S1 [file ECE3-10-10139-s001.docx]

**APPENDIX**


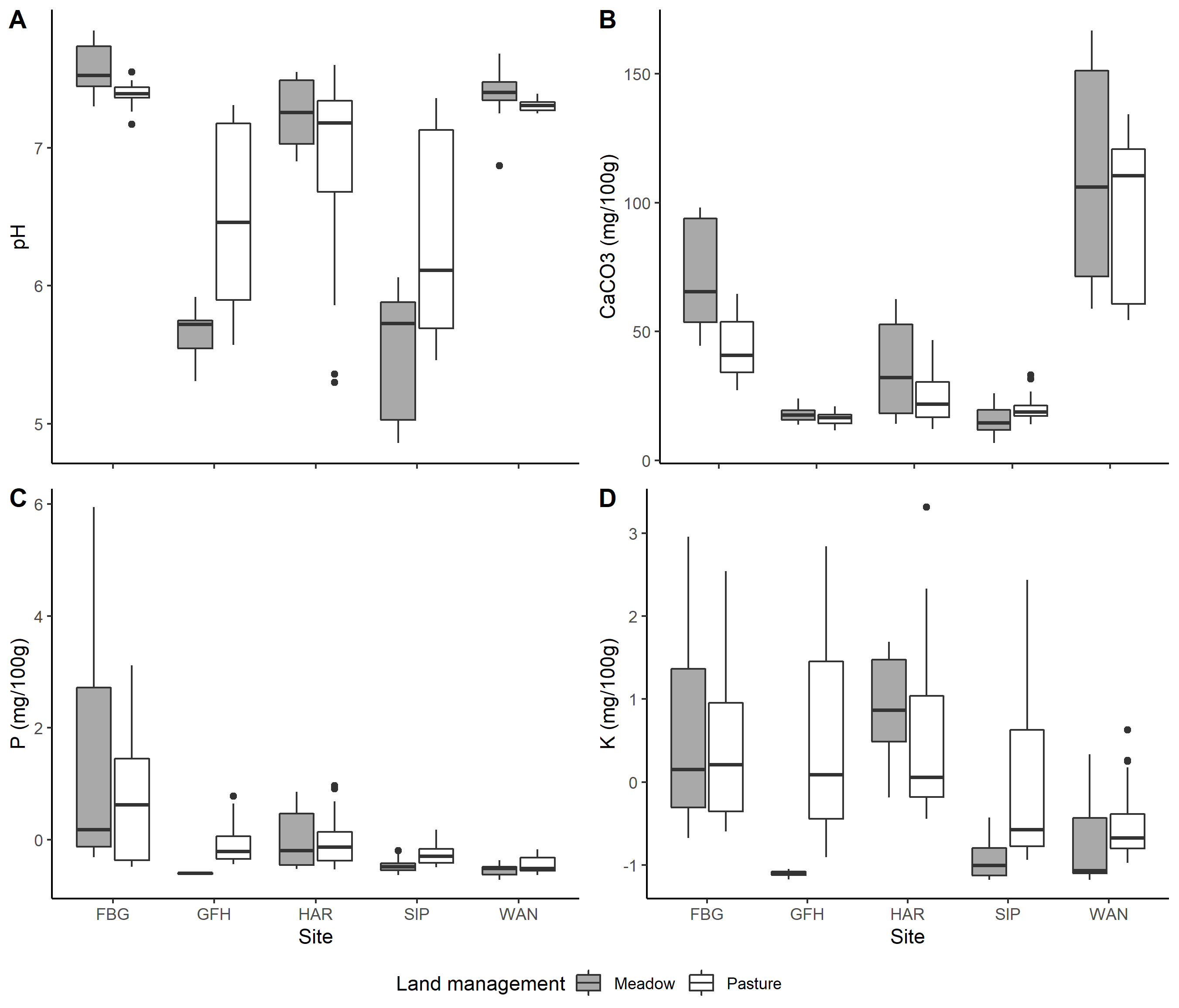


**Figure S1. Boxplots of soil properties across grassland land management (LM) types within and across study sites (Friedeburg FBG; Greifenhagen, GHF; Harsleben, HAR; Siptenfelde, SIP and Wanzleben, WAN). Site names refer to the closest large village, see Table 1 for further details. Soil parameters tested include the, pH concentration, pH; calcium carbonate, CaCO3; phosphorus, P and potassium, K. P and K are measured at plant available concentrations. The analyses per soil variable were performed as mixed effect models with interaction effect between LM types (meadow versus pasture) and site location. See Table A5 for further details and ANOVA results.**


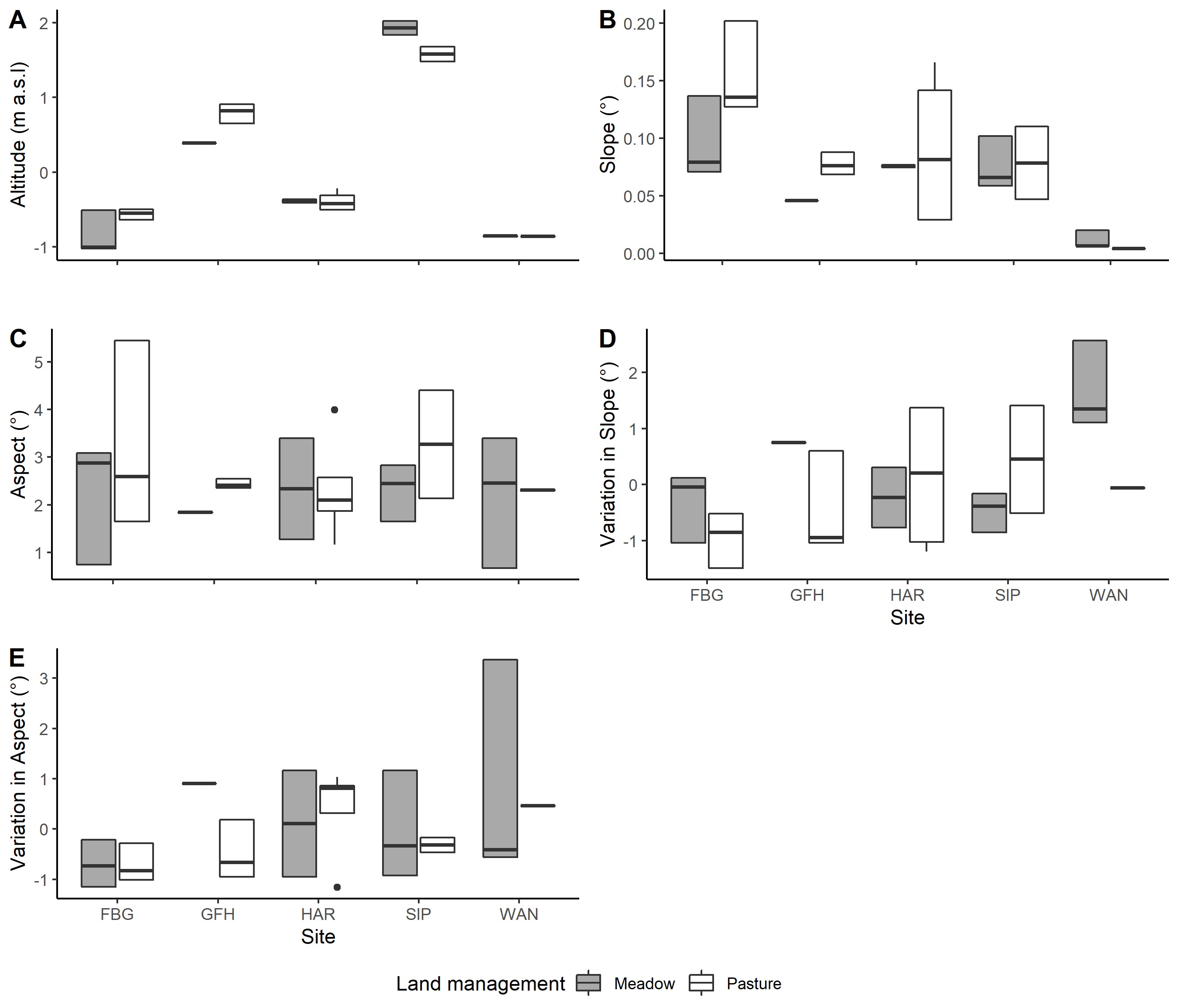


**Figure S2 Boxplots of landscape features across grassland land management (LM) types within and across study sites (Friedeburg FBG; Greifenhagen, GHF; Harsleben, HAR; Siptenfelde, SIP and Wanzleben, WAN). Site names refer to the closest large village, see Table 1 for further details. The analyses per landscape feature were performed as mixed effect models with interaction effect between LM types (meadow versus pasture) and site location, followed by a type III ANOVA. See Table A5 for further details and ANOVA results.**


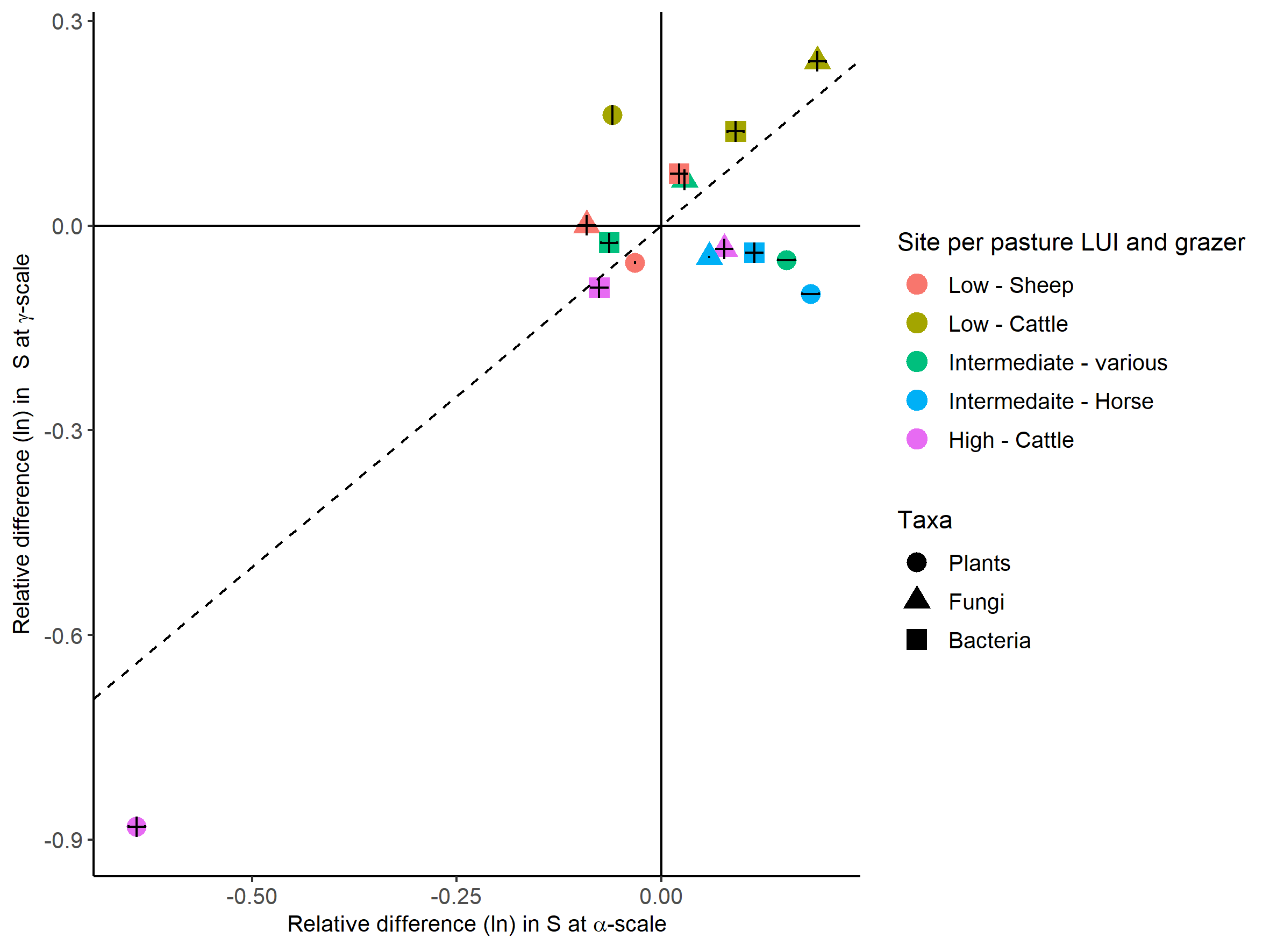


**Figure S3. Scale-dependent impact of land management (pasture versus meadow) observed as a change in the log response ratio (Relative difference (ln)) in species richness (S) at the α- (subplot-) and γ-scale (site-level) for above- (i.e. plants, circles) and belowground taxa (i.e. soil fungi and bacteria, triangles and squares, respectively). The log response ratios between management types were calculated with meadows as reference, thus positive values indicated that S is higher in pasture management. Horizontal and vertical bars indicate where there was a significant difference (p<0.05) in S between LM types based on the ANOVA and permutation tests, for α- and γ-scales respectively. The dashed 1:1 line indicates no scale-dependence. Sites are color coded according to pasture land use intensity (LUI) calculated as livestock units per hectare per annum, see Table 1 for more details.**


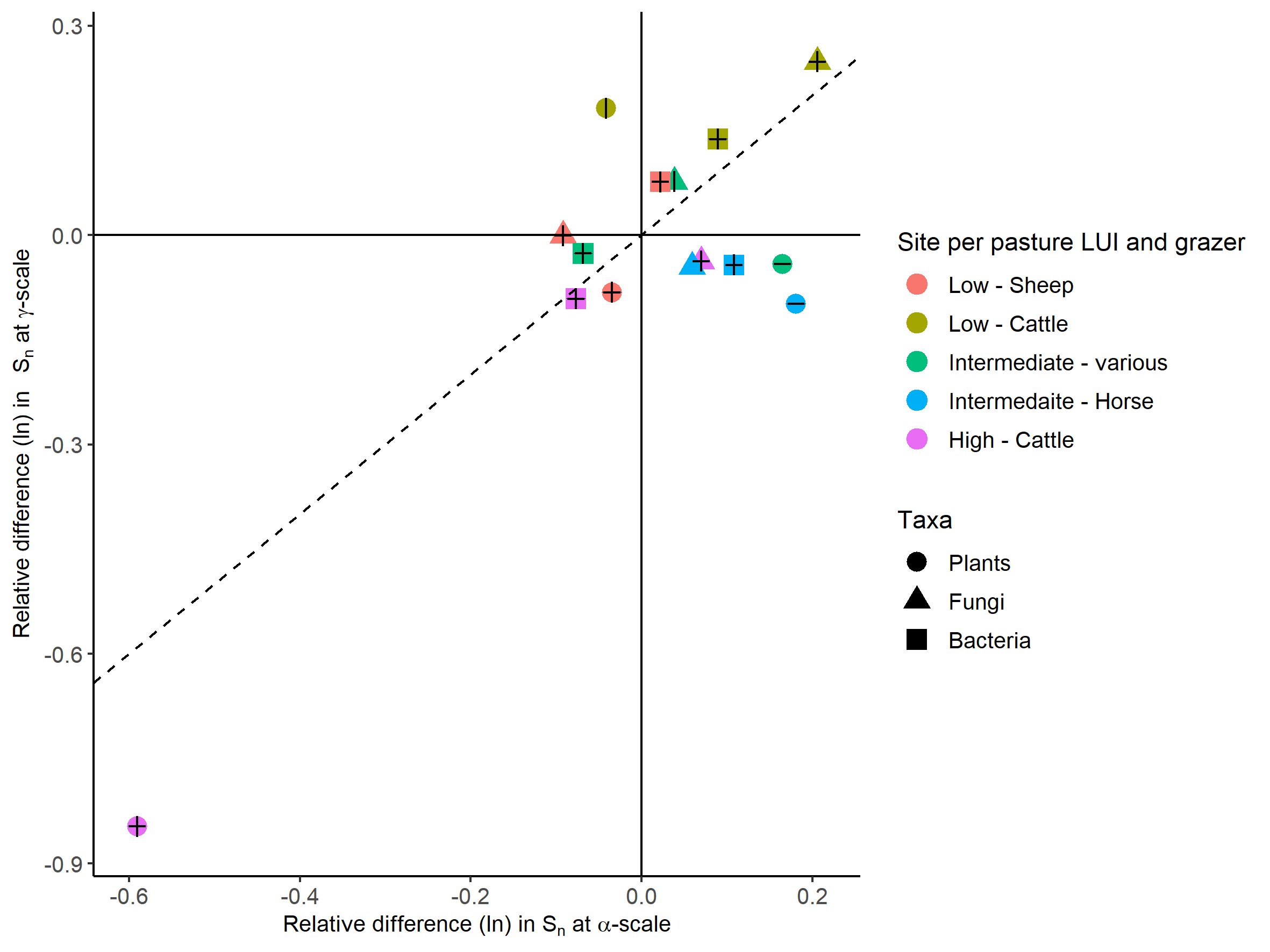


**Figure S4. Scale-dependent impact of land management (pasture versus meadow) observed as a change in the log response ratio (Relative difference (ln)) in rarefied species richness (S_n_) at the α- (subplot-) and γ-scale (site-level) for above- (i.e. plants, circles) and belowground taxa (i.e. soil fungi and bacteria, triangles and squares, respectively). The log response ratios between management types were calculated with meadows as reference, thus positive values indicated that S_n_ is higher in pasture management. Horizontal and vertical bars indicate where there was a significant difference (p<0.05) in S_n_ between LM types based on the ANOVA and permutation tests, for α- and γ-scales respectively. The dashed 1:1 line indicates no scale-dependence. Sites are color coded according to pasture land use intensity (LUI) calculated as livestock units per hectare per annum, see Table 1 for more details.**


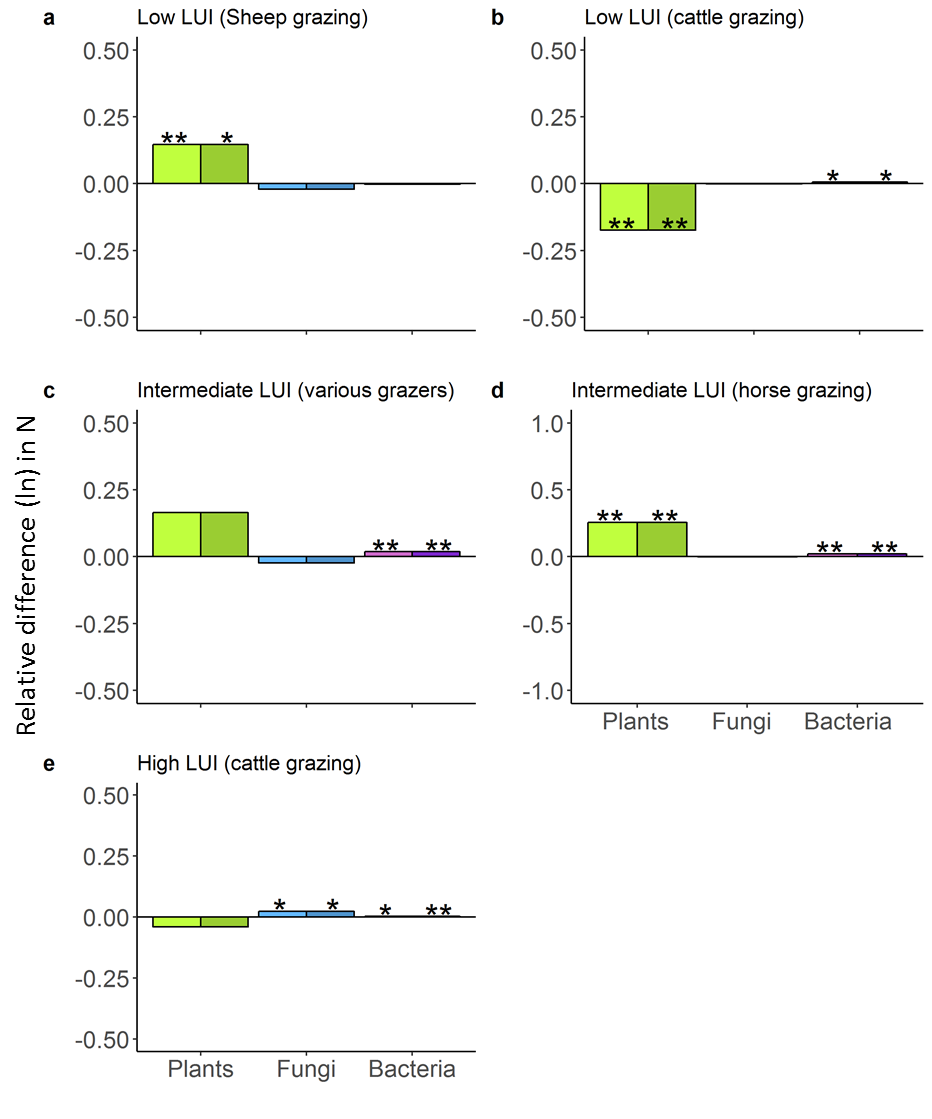

Relative difference (ln) in β-S_n_

**Figure S5. Scale-dependent impact of land management (pasture versus meadow) on the log response ratio (Relative difference (ln)) in density of individuals (N) for above- (i.e. plants, green) and belowground taxa (i.e. soil fungi and bacteria, blue and purple, respectively). The log response ratios between management types were calculated with meadows as reference at α- (subplot-, lighter hue) and γ-scale (site-level, darker hue) per taxa. Asterisks indicate significance differences (‘*’, p<0.05; ‘**’, p<0.01; ‘***’, p<0.001) between management types based on ANOVA and permutation tests, for α- and γ-scales respectively. Sites are labeled according to pasture land use intensity (LUI) calculated as livestock units per hectare per annum, see Table 1 for more details.**


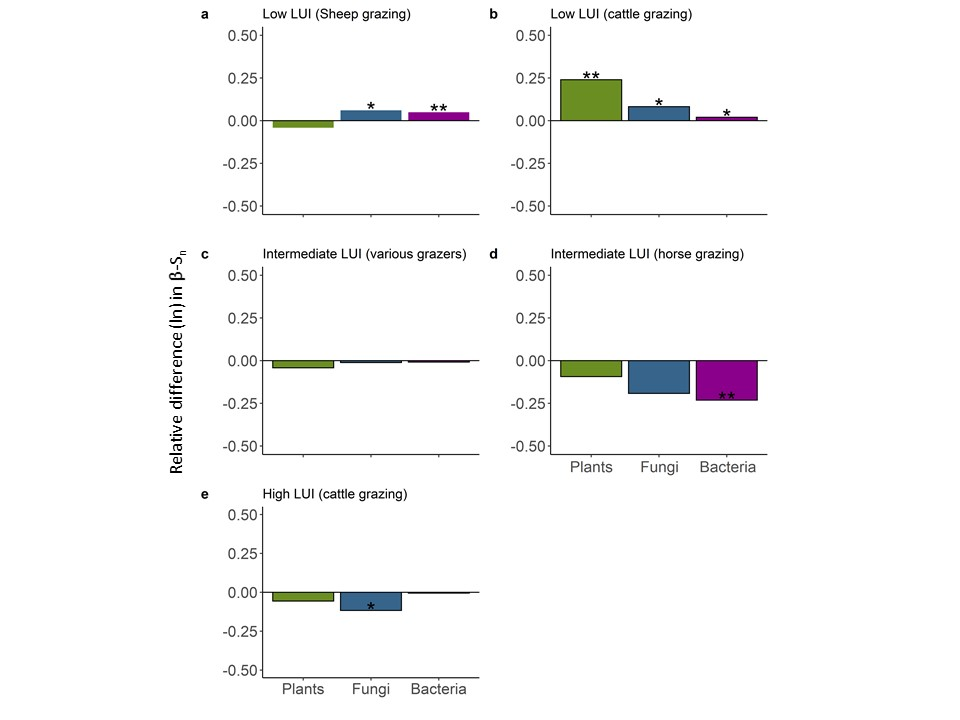


**Figure S6. Scale-dependent impact of land management (pasture versus meadow) on the log response ratio (Relative difference (ln)) in turnover of rare species (β-S_n_) for above- (i.e. plants, green) and belowground taxa (i.e. soil fungi and bacteria, blue and purple, respectively). The log response ratios between management types were calculated with meadows as reference, thus positive values indicate higher spatial aggregation in pastures. Asterisks indicated significant differences (‘*’, p<0.05; ‘**’, p<0.01; ‘***’, p<0.001) across management types based on permutation tests. Sites are labeled according to pastoral land use intensity (LUI) calculated as livestock units per hectare per annum. See Table 1 for more details on LUI per site.**

Table S1. Average soil chemical properties of the grasslands managed as meadows or pastures at the respective TERENO sites. Properties listed include the, pH value, pH; organic carbon to total nitrogen ratio, C:N; total organic carbon, TOC; total nitrogen, N; calcium carbonate, CaCO3; potassium, K; phosphorus, P. Nutrients are measured as mg/100g soil, with P and K as the plant available concentrations. Site names refer to the nearest large village. Asterisks’ indicate significance differences (‘*’, p<0.05; ‘**’, p<0.01; ‘***’, p<0.001) between management types based on a Welch t-test. See Table 1 for more site detail.

| Study site | Soil parameters | Meadow | | Pasture | | t-value | p-value |
| --- | --- | --- | --- | --- | --- | --- | --- |
|  |  | Mean | Standard error | Mean | Standard error |  |  |
| Harsleben | pH | 7.24 | 0.11 | 6.91 | 0.11 | 3.04 | ** |
|  | C:N | 8.36 | 0.13 | 9.59 | 0.13 | -9.60 | *** |
|  | TOC | 17.06 | 0.63 | 21.84 | 0.63 | -7.60 | *** |
|  | TN | 2.04 | 0.07 | 2.28 | 0.07 | -3.58 | *** |
|  | CaCO_3_ | 35.05 | 4.34 | 23.94 | 4.34 | 2.56 | * |
|  | K | 36.65 | 3.00 | 30.06 | 3.00 | 2.20 | * |
|  | P | 5.97 | 1.16 | 5.97 | 1.16 | 0.00 | ns |
| Siptenfelde | pH | 5.54 | 0.18 | 6.34 | 0.18 | -4.41 | *** |
|  | C:N | 9.10 | 0.25 | 9.26 | 0.25 | -0.65 | ns |
|  | TOC | 25.11 | 1.86 | 37.02 | 1.86 | -6.41 | *** |
|  | TN | 2.69 | 0.13 | 4.00 | 0.13 | -9.88 | *** |
|  | CaCO_3_ | 15.88 | 1.51 | 20.34 | 1.51 | -2.94 | ** |
|  | K | 8.96 | 3.68 | 23.46 | 3.68 | -3.94 | *** |
|  | P | 1.85 | 0.45 | 3.81 | 0.45 | -4.37 | *** |
| Friedeburg | pH | 7.59 | 0.03 | 7.39 | 0.03 | 5.83 | *** |
|  | C:N | 10.52 | 0.20 | 9.98 | 0.20 | 2.74 | * |
|  | TOC | 25.04 | 1.62 | 22.44 | 1.62 | 1.61 | ns |
|  | TN | 2.42 | 0.17 | 2.25 | 0.17 | 1.00 | ns |
|  | CaCO_3_ | 71.58 | 4.19 | 44.59 | 4.19 | 6.44 | *** |
|  | K | 30.36 | 3.54 | 29.05 | 3.54 | 0.37 | ns |
|  | P | 18.87 | 4.19 | 13.87 | 4.19 | 1.19 | ns |
| Wanzleben | pH | 7.41 | 0.03 | 7.31 | 0.03 | 3.63 | *** |
|  | C:N | 10.97 | 0.22 | 10.32 | 0.22 | 3.01 | ** |
|  | TOC | 47.02 | 2.69 | 49.39 | 2.69 | -0.88 | ns |
|  | TN | 4.43 | 0.30 | 4.79 | 0.30 | -1.18 | *** |
|  | CaCO_3_ | 109.60 | 8.70 | 97.70 | 8.70 | 1.37 | ns |
|  | K | 11.44 | 1.67 | 15.25 | 1.67 | -2.29 | * |
|  | P | 1.08 | 0.30 | 2.02 | 0.30 | -3.16 | ** |
| Greifenhagen | pH | 5.67 | 0.12 | 6.49 | 0.12 | -6.66 | *** |
|  | C:N | 7.41 | 0.10 | 8.27 | 0.10 | -8.46 | *** |
|  | TOC | 15.05 | 0.77 | 22.90 | 0.77 | -10.26 | *** |
|  | TN | 2.04 | 0.09 | 2.76 | 0.09 | -8.49 | *** |
|  | CaCO_3_ | 18.02 | 1.07 | 16.48 | 1.07 | 1.44 | ns |
|  | K | 6.65 | 2.88 | 29.63 | 2.88 | -7.99 | *** |
|  | P | 0.51 | 0.57 | 5.41 | 0.57 | -8.52 | *** |

Table S2. Analysis of Deviance for linear mixed effects models (random intercept for site) performed on the (a) soil properties and (b) landscape features across land management (LM) types (meadow versus pasture) and within sites, including the interaction of LM and site. Soil parameters tested include the, pH values, pH; calcium carbonate, CaCO3; phosphorus, P and potassium, K. Landscape features tested include the grasslands altitude, slope and coefficient of variation in slope (Slope cv) and aspect and coefficient of variation thereof (Aspect cv). Results report type III Wald chi-square test results including the Chi^2^ value, degrees of freedom (Df) and the p-value of the Chi^2^ test (p-chi^2^). Asterisks’ indicate significance differences: 0, ‘***’; 0.001, ‘**’; 0.01, ‘*’; 0.05, ‘.’). For more detail on the sites, see Table 1.


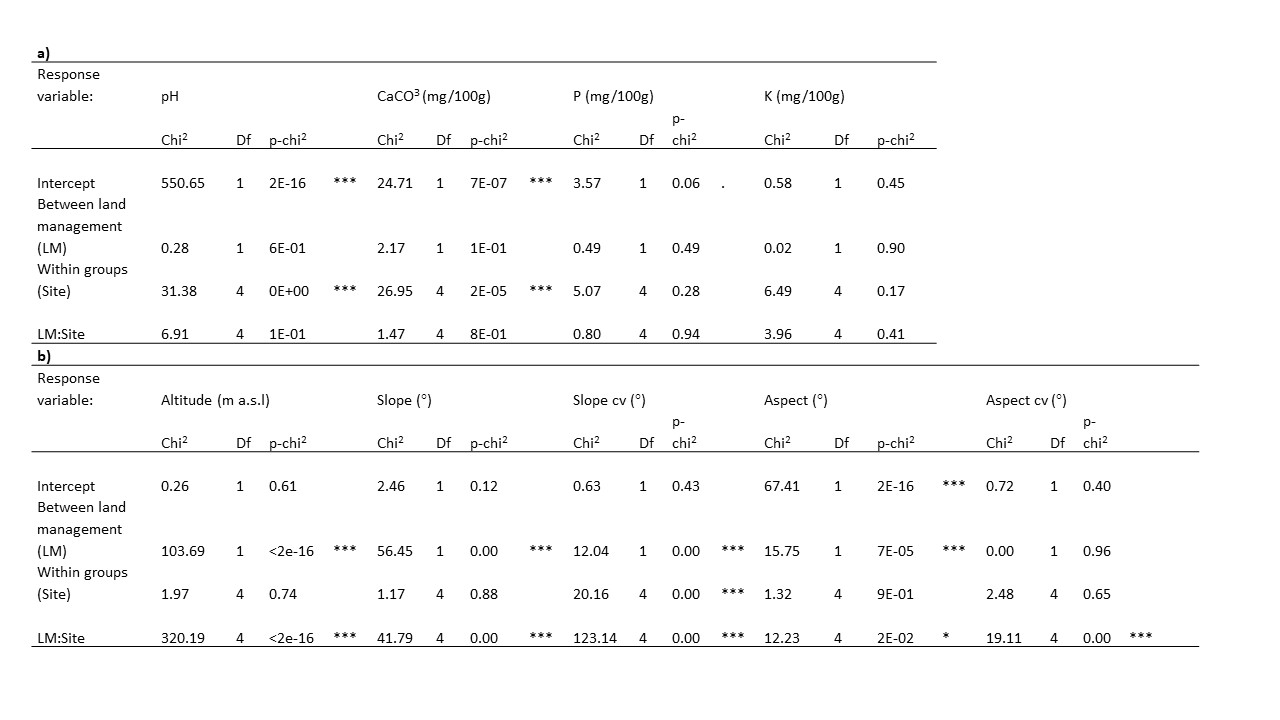


**Table S3. Overview of the most abundant species per taxonomic group (plants, fungi and bacteria) across the TERENO sites. The top 5 rank abundant species are listed per site-land management combination, with the top 5 indicated in bold. (a) Plant species are reported as proportional % cover and sum of % cover of the top ranked species. (b) Fungi and (c) bacteria where recorded to operational taxonomic units (OTU; for bacteria these are used as species equivalent, while for fungi these could be associated with specific species. These are reported as proportional and total OTU reads (used as a proxy for abundance) for the top ranked species per taxa. Site names refer to the nearest large village.**


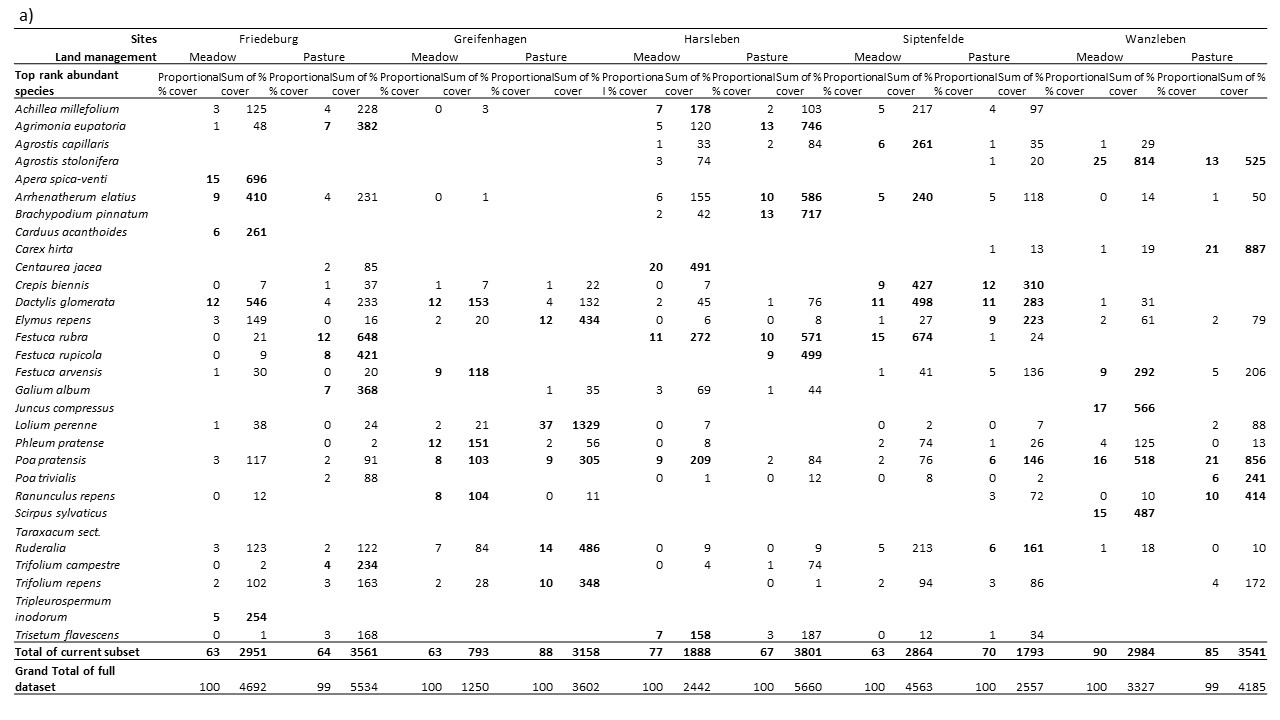


(b)

|  | **Sites** | Friedeburg | | | | Greifenhagen | | | | Harsleben | | | | Siptenfelde | | | | Wanzleben | | | |
| --- | --- | --- | --- | --- | --- | --- | --- | --- | --- | --- | --- | --- | --- | --- | --- | --- | --- | --- | --- | --- | --- |
|  | **Land management** | Meadow | | Pasture | | Meadow | | Pasture | | Meadow | | Pasture | | Meadow | | Pasture | | Meadow | | Pasture | |
| **Species** | **Phylum** | Proportion | Sum | Proportion | Sum | Proportion | Sum | Proportion | Sum | Proportion | Sum | Proportion | Sum | Proportion | Sum | Proportion | Sum | Proportion | Sum | Proportion | Sum |
| *Mortierella minutissima* | Zygomycota | **6** | **27143** | **4** | **16576** | **9** | **12433** | **5** | **21431** | **7** | **19559** | 1 | 4117 | **4** | **18425** | **9** | **25445** | **6** | **24819** | **8** | **32328** |
| *Exophiala equina* | Ascomycota | **3** | **13752** | **2** | **9162** | **2** | 3395 | **7** | **30773** | **4** | **10202** | **3** | **14634** | 1 | 2675 | 2 | 4208 | 1 | 5150 | 0 | 494 |
| *Cryptococcus terreus* | Basidiomycota | 2 | 8452 | **2** | **8422** | 3 | 4862 | **6** | **25307** | **6** | **18167** | 2 | 12460 | 1 | 4520 | 1 | 3722 | 0 | 44 |  |  |
| *Cryptococcus terricola* | Basidiomycota | 0 | 50 |  |  | **4** | **5783** | 2 | 6557 | 0 | 62 | 0 | 1901 | **3** | **13778** | **3** | **7891** | 0 | 1 | 0 | 2 |
| *Preussia flanaganii* | Ascomycota | 1 | 2142 | 2 | 6994 | 0 | 434 | **4** | **15498** | **6** | **16843** | 1 | 6827 | 0 | 738 | **2** | **6957** | 0 | 921 | 2 | 6461 |
| *Mortierella alpina* | Zygomycota | **2** | **9454** | 1 | 2917 | 1 | 753 | 2 | 7364 | 1 | 1386 | 1 | 3172 | 1 | 4764 | **2** | **6958** | 0 | 35 | 0 | 17 |
| *Mortierella exigua* | Zygomycota | 1 | 2629 | 1 | 4545 | **4** | **5196** | 1 | 4391 | 1 | 3433 | 0 | 1992 | 0 | 840 | 1 | 3131 | 2 | 9072 | **3** | **13184** |
| *Mortierella elongata* | Zygomycota | **4** | **17943** | 2 | 6886 | 0 | 41 | 2 | 9325 | 2 | 6740 | 0 | 930 | 0 | 9 | 0 | 382 |  |  |  |  |
| *Camarophyllopsis hymenocephala* | Basidiomycota |  |  | 0 | 25 |  |  | 0 | 2 | 0 | 2 | **5** | **30826** |  |  |  |  |  |  |  |  |
| *Agaricales spp.* | Basidiomycota |  |  |  |  |  |  |  |  |  |  |  |  |  |  |  |  | **6** | **24277** | **3** | **11947** |
| *Ascomycota spp.* | Ascomycota |  |  |  |  |  |  |  |  |  |  |  |  |  |  |  |  | **8** | **34487** |  |  |
| *Cryptococcus spp.* | Basidiomycota | 0 | 2 |  |  | 4 | **5551** | 0 | 1097 | 0 | 3 | 0 | 316 | 2 | 7197 | 1 | 3622 | 0 | 1 | 0 | 1 |
| *Agaricales spp.* | Basidiomycota |  |  |  |  |  |  |  |  |  |  | **4** | **24405** |  |  |  |  |  |  |  |  |
| *Inocybe griseovelata* | Basidiomycota | **7** | **29127** | 0 | 3 |  |  | 0 | 1 |  |  |  |  |  |  |  |  | 0 | 9 |  |  |
| *Ascomycota spp.* | Ascomycota |  |  | 0 | 640 |  |  | 0 | 233 | **4** | **10933** | 2 | 10649 |  |  |  |  |  |  |  |  |
| *Clavariaceae spp.* | Basidiomycota | 0 | 1 | 0 | 4 | 1 | 929 | 0 | 1954 | 0 | 312 | 0 | 1903 | **4** | **16028** | 0 | 375 | 1 | 2747 |  |  |
| *Clavaria spp.* | Basidiomycota |  |  |  |  |  |  |  |  |  |  |  |  | **4** | **16094** | 0 | 89 |  |  |  |  |
| *Hygrocybe nigrescens* | Basidiomycota |  |  |  |  |  |  | 0 | 1 |  |  | **3** | **15169** | 2 | 8819 |  |  |  |  |  |  |
| *Unclassified fungi* | Unclassified fungi | 0 | 217 | **2** | **8224** |  |  |  |  | 0 | 1 | 2 | 13759 |  |  |  |  |  |  |  |  |
| *Mortierella spp.* | Zygomycota | 0 | 1 | 0 | 1 | **9** | **12990** |  |  |  |  | 0 | 82 | 1 | 2444 | 0 | 330 |  |  |  |  |
| *Cuphophyllus spp.* | Basidiomycota |  |  |  |  |  |  |  |  |  |  | **3** | **18430** |  |  |  |  |  |  |  |  |
| *Ascomycota spp.* | Ascomycota |  |  | **3** | **14266** |  |  |  |  | 0 | 256 | 0 | 802 |  |  |  |  |  |  |  |  |
| *Funneliformis geosporum* | Glomeromycota | 0 | 17 | 0 | 102 |  |  | 0 | 36 | 0 | 264 | 0 | 4 | 0 | 9 | 0 | 487 | 1 | 3419 | **3** | **13786** |
| *Ascomycota spp.* | Ascomycota |  |  |  |  |  |  |  |  |  |  |  |  |  |  |  |  | **4** | **16618** | 0 | 141 |
| *Agaricales spp.* | Basidiomycota |  |  |  |  |  |  | 0 | 14 |  |  | 0 | 4 | **4** | **17682** | 0 | 1 |  |  |  |  |
| *Mortierella spp.* | Zygomycota |  |  |  |  |  |  | 0 | 17 |  |  |  |  |  |  | 0 | 56 | 0 | 269 | **3** | **12465** |
| *Agaricus campestris* | Basidiomycota |  |  |  |  | 0 | 6 | **2** | **10095** |  |  |  |  |  |  | 0 | 9 |  |  |  |  |
| *Phoma herbarum* | Ascomycota |  |  |  |  |  |  |  |  |  |  |  |  |  |  |  |  | **2** | **10614** | 0 | 1 |
| *Geoglossales spp.* | Ascomycota |  |  |  |  |  |  |  |  |  |  |  |  |  |  | **3** | **8385** |  |  |  |  |
| **Total of current subset** | | **25** | **110930** | **18** | **78767** | **36** | **52373** | **30** | **134096** | **30** | **88163** | **28** | **162382** | **27** | **114022** | **25** | **72048** | **30** | **132483** | **22** | **90827** |
| **Grand Total of filtered dataset** | | 90 | 437258 | 91 | 427315 | 93 | 144550 | 92 | 444239 | 92 | 292648 | 92 | 573047 | 91 | 426929 | 89 | 284368 | 93 | 442542 | 93 | 412859 |


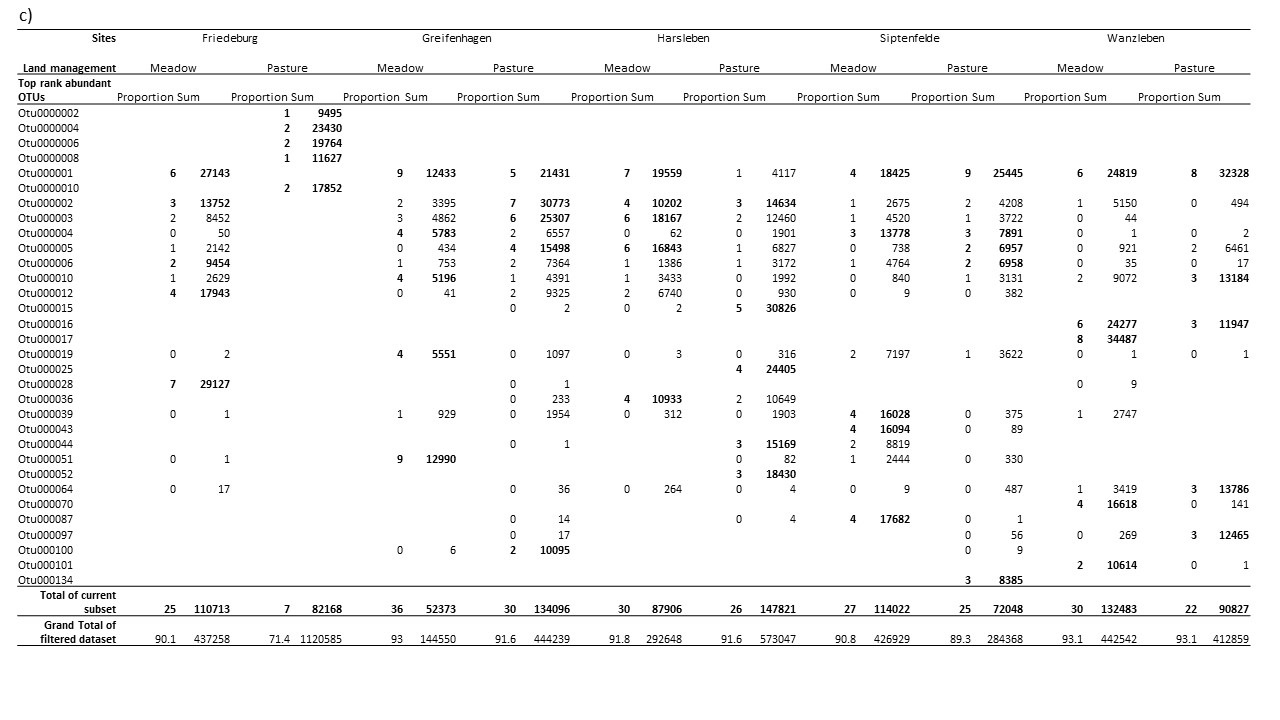


**Table S4. Biodiversity metrics used to investigate scale-dependency of land management (pasture versus meadow) responses in semi-natural grasslands. Table adapted from Chase *et al*. (2018).**

| Metric | Definition | Interpretation |
| --- | --- | --- |
| N | Total number of individuals | Measure of density of individuals. N scales roughly linearly with area. |
| α-S, γ-S | Observed richness of species from α-scale (average of observations at subplot-level per LM) and from γ-scale (sum across all observations per LM) | Number of species at local scale (= α-diversity) and large scale (= γ-diversity) |
| α-S_n_, γ-S_n_ | The expected richness for *n* randomly sampled individuals (Hurlbert 1971). Can be calculated from α- or γ-scale | Estimate of richness at α- or γ-scale after controlling for differences due to aggregation and number of individuals [i.e. only reflects species abundance distribution (SAD)] |
| α-PIE, γ-PIE | Probability of interspecific encounter  (S_n = 2_ - S_n = 1_, Hurbert 1971; Olszweski 2004). Can be calculated from α- and γ-scale. | Measure of evenness at α- or γ-scale that is quantified by the slope at the base of the rarefaction curve; sensitive to common species. |
| α-S_PIE_, γ-S_PIE_ | Equally abundant species needed to yield a probability of encounter (PIE) at α-or γ-scale (Jost 2006) (= 1/(1 - PIE)). | Effective number of species of PIE (= 1 - Simpson diversity index); measured at α- or γ-scale |
| β-S | Ratio of total treatment γ-S and average plot α-S (Whittaker 1960) | More species turnover results in larger β-S due to increases in spatial aggregation, N, and/or unevenness of the SAD |
| β-S_n_ | Ratio of the total treatment γ-S_n_ and α-S_n_ | Like β-S but emphasizes aggregation due to common and rare species |
| β-S_PIE_ | Ratio of total treatment γ-S_PIE_ and α-S_PIE_ (Jost, 2007) | Like β-S but emphasizes aggregation due to common species only |

Table S5. Absolute difference between land management (pasture versus meadow) in the density of individuals (N), species richness (S), rarefied species richness (S_n_) and evenness (S_PIE_) between management types. Differences were calculated with meadows as reference, and are presented with p-values derived from ANOVA and permutation tests, for the respective scales. Significant values (p<0.05) are marked in bold. Site names refer to the nearest large village along with pasture land use intensity (LUI) calculated as livestock units per hectare per annum, see Table 1 for more details.

| **a) Alpha diversity** | |  |  |  |  |  |  |
| --- | --- | --- | --- | --- | --- | --- | --- |
| Site | Index | Plants | | Fungi | | Bacteria | |
|  |  | Difference (D-bar) | p-value | Difference (D-bar) | p-value | Difference (D-bar) | p-value |
| HAR | N | **19.40** | **0.01** | -306.23 | 0.16 | -61.80 | 0.31 |
|  | S | -0.57 | 0.13 | **-36.50** | **0.02** | **60.65** | **0.02** |
|  | S_n_ | **-0.56** | **0.03** | **-35.70** | **0.02** | **61.48** | **0.02** |
|  | S_PIE_ | -0.27 | 0.69 | **-11.98** | **0.01** | 2.66 | 0.68 |
| SIP | N | **-24.25** | **0.01** | -12.57 | 0.42 | **234.45** | **0.01** |
|  | S | -0.97 | 0.35 | **85.85** | **0.01** | **270.20** | **0.01** |
|  | S_n_ | -0.64 | 0.45 | **85.97** | **0.01** | **264.32** | **0.01** |
|  | S_PIE_ | -0.88 | 0.27 | **20.02** | **0.01** | **94.63** | **0.01** |
| FBG | N | 28.07 | 0.14 | -331.43 | 0.42 | **690.47** | **0.01** |
|  | S | **2.30** | **0.03** | 11.00 | 0.34 | **-183.83** | **0.01** |
|  | S_n_ | **2.31** | **0.01** | **13.00** | **0.20** | **-195.88** | **0.01** |
|  | S_PIE_ | **2.79** | **0.01** | **11.80** | **0.02** | **-55.24** | **0.01** |
| WAN | N | **34.03** | **0.01** | -2.43 | 0.61 | **829.57** | **0.01** |
|  | S | **1.87** | **0.01** | 15.67 | 0.43 | **319.67** | **0.01** |
|  | S_n_ | **1.65** | **0.01** | 15.81 | 0.44 | **297.29** | **0.01** |
|  | S_PIE_ | **2.55** | **0.01** | 4.43 | 0.33 | 26.70 | 0.19 |
| GFH | N | -4.93 | 0.51 | **352.97** | **0.01** | **132.40** | **0.01** |
|  | S | **-7.33** | **0.01** | **30.93** | **0.03** | **-216.73** | **0.01** |
|  | S_n_ | **-6.47** | **0.01** | **27.54** | **0.03** | **-218.78** | **0.01** |
|  | S_PIE_ | **-4.10** | **0.01** | **2.78** | **0.05** | **-125.99** | **0.01** |
| **b) Gamma diversity** | | |  |  |  |  |  |
| Site | Index | Plants | | Fungi | | Bacteria | |
|  |  | Difference (D-bar) | p-value | Difference (D-bar) | p-value | Difference (D-bar) | p-value |
| **HAR** | N | **388.00** | **0.01** | -6124.50 | 0.22 | -1236.00 | 0.28 |
|  | S | -3.33 | 0.17 | **0.83** | **0.02** | **409.33** | **0.01** |
|  | S_n_ | **-4.75** | **0.04** | **-0.98** | **0.03** | **409.90** | **0.01** |
|  | S_PIE_ | -2.64 | 0.10 | -3.30 | 0.11 | **27.29** | **0.01** |
| **SIP** | N | **-485.00** | **0.01** | -251.33 | 0.38 | **4689.00** | **0.01** |
|  | S | **7.33** | **0.04** | **357.33** | **0.01** | **794.00** | **0.01** |
|  | S_n_ | **8.04** | **0.01** | **358.39** | **0.01** | **791.31** | **0.01** |
|  | S_PIE_ | **3.41** | **0.03** | 4.77 | 0.72 | **187.90** | **0.01** |
| **FBG** | N | 842.00 | 0.09 | -9943.00 | 0.37 | **20714.00** | **0.01** |
|  | S | -3.00 | 0.53 | **101.00** | **0.01** | **-129.00** | **0.01** |
|  | S_n_ | -2.36 | 0.49 | **110.06** | **0.01** | **-133.73** | **0.01** |
|  | S_PIE_ | 5.07 | 0.19 | **50.24** | **0.01** | **-101.64** | **0.01** |
| **WAN** | N | **1021.00** | **0.01** | -73.00 | 0.61 | **24887.00** | **0.01** |
|  | S | -4.00 | 0.50 | -51.00 | 0.14 | **-218.00** | **0.01** |
|  | S_n_ | -3.72 | 0.50 | -49.89 | 0.14 | **-234.27** | **0.01** |
|  | S_PIE_ | 0.54 | 0.54 | 9.34 | 0.49 | **-116.96** | **0.01** |
| **GFH** | N | -49.33 | 0.50 | **3529.67** | **0.01** | **1324.00** | **0.01** |
|  | S | **-21.67** | **0.01** | **-34.00** | **0.01** | **-427.00** | **0.01** |
|  | S_n_ | **-20.01** | **0.01** | **-36.86** | **0.01** | **-427.81** | **0.01** |
|  | S_PIE_ | **-8.88** | **0.01** | 6.53 | 0.25 | **-136.70** | **0.01** |
| **c) Beta diversity** | |  |  |  |  |  |  |
| Site | Index | Plants | | Fungi | | Bacteria | |
|  |  | Difference (D-bar) | p-value | Difference (D-bar) | p-value | Difference (D-bar) | p-value |
| **HAR** | S | -0.14 | 0.72 | **0.46** | **0.01** | **0.11** | **0.01** |
|  | S_n_ | -0.07 | 0.07 | **0.13** | **0.03** | **0.06** | **0.01** |
|  | S_PIE_ | -0.72 | 0.06 | **3.35** | **0.01** | **0.05** | **0.02** |
| **SIP** | S | **0.67** | **0.01** | 0.16 | 0.14 | **0.08** | **0.02** |
|  | S_n_ | **0.41** | **0.01** | **0.17** | **0.02** | **0.03** | **0.01** |
|  | S_PIE_ | **0.74** | **0.01** | **-2.02** | **0.01** | **0.17** | **0.01** |
| **FBG** | S | -0.58 | 0.06 | **0.21** | **0.05** | **0.07** | **0.01** |
|  | S_n_ | -0.09 | 0.51 | -0.03 | 0.53 | -0.01 | 0.43 |
|  | S_PIE_ | **1.26** | **0.05** | 0.27 | 0.52 | **-0.13** | **0.01** |
| **WAN** | S | **-1.21** | **0.01** | **-1.21** | **0.02** | **-0.34** | **0.01** |
|  | S_n_ | -0.18 | 0.14 | -0.59 | 0.14 | **-0.29** | **0.01** |
|  | S_PIE_ | **-1.34** | **0.01** | -1.64 | 0.10 | **-0.50** | **0.01** |
| **GFH** | S | **-0.55** | **0.01** | **-0.28** | **0.01** | -0.02 | 0.09 |
|  | S_n_ | -0.08 | 0.56 | **-0.20** | **0.01** | -0.01 | 0.27 |
|  | S_PIE_ | **-0.54** | **0.01** | 0.29 | 0.65 | 0.02 | 0.48 |
